# Protocol for assessing antibody-mediated cellular phagocytosis of lymphoma cells by human and murine macrophages *in vitro*

**DOI:** 10.1016/j.xpro.2025.104126

**Published:** 2025-10-08

**Authors:** Anna C. Beielstein, Stuart Blakemore, Christian P. Pallasch

**Affiliations:** 1 Department I of Internal Medicine, Centre for Integrated Oncology (CIO) Aachen-Bonn-Cologne-Duesseldorf, University Hospital Cologne, 50937 Cologne, Germany; 2 Cologne Excellence Cluster for Cellular Stress Responses in Ageing-Associated Diseases (CECAD), University of Cologne, 50931 Cologne, Germany; 3 Centre for Molecular Medicine Cologne (CMMC), University of Cologne, 50937 Cologne, Germany

**Keywords:** antibody, Cancer, cell culture, Immunology, model Organisms

## Abstract

Antibody-dependent phagocytosis is an important process of cell engulfment and degradation by macrophages that holds therapeutical importance in the context of chemo-immunotherapy in, for example, lymphoma. Here, we present an *in vitro* protocol for determining the antibody-dependent phagocytosis of lymphoma cells by different types of macrophages under different treatment conditions. We describe steps for generating mature macrophages from different sources, usage of different clinically established antibodies, and elucidating the driving cell type of the observed changes in phagocytosis.

For complete details on the use and execution of this protocol, please refer to Beielstein et al.^1^

## Highlights

• Identify the antibody-dependent phagocytosis of macrophages from different origins
• Determine the susceptibility toward phagocytosis of lymphoma cells
• Evaluate the influence of agents on the antibody-dependent phagocytosis
• Determine the leading cell type causing changed phagocytic activity

## Abstract (editor highlights)

Publisher’s note: Undertaking any experimental protocol requires adherence to local institutional guidelines for laboratory safety and ethics.

## Graphical abstract

**Figure:**
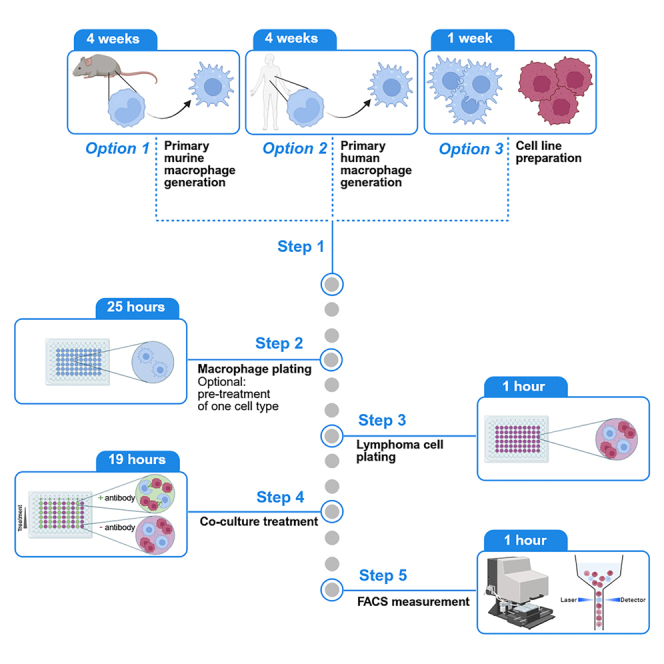

## Before you begin

In the treatment of lymphoma, the introduction of therapeutic antibodies has led to a significant increase in therapy response and cure rates.^2^ We have shown that a certain immune cell – the macrophage – is crucial for the success of antibody treatment in lymphoma.^3^ Macrophages actively phagocytose antibody-labeled lymphoma cells leading to profound reduction of disease burden and lymphoma eradication. However, it has also been shown that lymphoma cells directly impair macrophages via various mechanisms, causing a lymphoma-sustaining and less phagocytic macrophage phenotype.^4^^,^^5^^,^^6^

For a better understanding and a modification of this interplay, we developed an assay for the observation of the antibody-dependent phagocytosis of lymphoma cells by macrophages.

The protocol below describes the steps for using a lymphoma cell line derived from humanized lymphoma mouse model or primary human chronic lymphocytic leukemia (CLL) cells, which allows the use of clinically implemented antibodies in the phagocytosis assay. However, other lymphoma cell lines or primary murine lymphoma cells and different antibodies with murine epitopes might be used. But the researcher has to keep in mind that antibody treatment can lead to clustering of the targeted cells, causing invalid flow cytometer measurement or at least also flow cytometer clogging. The protocol describes the steps for different sources of macrophages (cell line, primary human peripheral blood-derived macrophages, primary murine bone marrow-derived macrophages). We have also used THP1 cells as a different cell line and primary murine peritoneal macrophages. The researcher has to keep in mind that for every change of a cell type in the protocol, the ratio between macrophages and lymphoma cells has to be evaluated for an adequate basal phagocytosis rate.

We have investigated the antibody-dependent phagocytosis of macrophages under metabolic modulation.^1^ We have also used the technique to investigate the influence of immune-checkpoint modulation^7^ and kinase inhibitors.^8^ Several more fields of macrophage or lymphoma cell modulation are feasible and the assay is - within certain limitations - scalable for screening of defined compound libraries.

### Innovation

With this protocol, the evaluation of antibody-dependent phagocytosis of lymphoma cells by macrophages by flow cytometer analysis is performed. Using a humanized lymphoma cell line as target cell enables the use of clinically implemented antibodies, facilitating research closer to actual clinical standards than artificial lab work. With the adaptation of the protocol to macrophages from different sources (cell line, primary murine cells, primary human cells), we open up a wide field of application. The used materials and the readout by flow cytometer measurement allow research without high costs or rare equipment.

### Institutional permissions

The use of cells extracted from mice were approved by local ethical review (LANUV (Landesamt für Natur, Umwelt und Verbraucherschutz Nordrhein-Westfalen)) and were carried out under the authority of Michael Michalik M. Sc., (University Hospital Cologne, Translational Research for Infectious Diseases and Oncology (TRIO), Robert-Koch-Straße 21, 50931 Cologne, Germany) project license. The use of healthy donor cells and primary chronic lymphocytic leukemia patient cells was approved by the ethical commission of the medical faculty of the University of Cologne (reference no. 13-091).

All experiments conform to the relevant regulatory standards.

If you want to perform the protocol using primary murine macrophages or primary lymphoma patient cells, you need to acquire permissions for performing animal experiments by local ethical review and for performing experiment with primary patient cells by ethical commission.

### Preparation

#### Preparation working with primary murine macrophages

**Timing: at least 4 weeks**

1. Produce feeder media for later supply of differentiating macrophage progenitor cells.
  a. Plate out the murine fibroblast cell line L-929 on a 10 cm dish in non-supplemented RPMI 1640 media.
    ***Note:*** The conditioned media of L-929 cells contains growth factors, cytokines and others, which are particularly suitable for the differentiation of macrophages. Therefore, we recommend to use the conditioned media of this certain cell line.
  b. When the L-929 cells are dense, change the media to fresh non-supplemented RPMI 1640 media.
  c. Incubate the L-929 cells.
  d. Collect the media of the L-292 cell dishes.
    i. After 1 week (further referred as “feeder media 1”).
    ii. After 3 weeks (further referred as “feeder media 2”).
      ***Note:*** Do not change the media until collecting day after performing step b.
  e. Centrifuge the media for 5 min at 300 *g* at 20°C to collect remaining cells in a pellet.
  f. Pipet the supernatant to a new tube to discharge the remaining cells.
  g. Store the feeder media at −20°C until further use.
2. Collect murine macrophage progenitor cells.
  a. Sacrifice mice.
  b. Dissect the femur.
  c. Flush the femur with 5 mL non-supplemented DMEM media.
  d. Centrifuge the cells at 360 *g* for 8 min at 4°C.
  e. Discard the supernatant.
  f. Add 2 mL Ammonium-Chloride-Potassium (ACK) lysis buffer to the cell pellet for 2 min to lyse erythrocytes.
  g. Add 50 mL of cold PBS to stop the reaction.
  h. Centrifuge the cells at 300 *g* for 5 min at 4°C.
  i. Discard the supernatant.
  j. Re-suspend the cells in 20 mL DMEM media supplemented with 10% fetal calf serum (FCS) + 1% Penicillin/Streptomycin.
  k. Incubate the cells for 24 h on two 10 cm cell culture plates.
  l. Collect the non-adherent cells and count them.
3. Differentiate the monocytes into macrophages.
  a. Plate out 6 × 10^5^ cells /mL in 5 mL DMEM media supplemented with 10% FCS + 1% Penicillin/Streptomycin on a 10 cm cell plate.
  b. Add 750 μL feeder media 1 and 750 μL feeder media 2.
  c. Incubate the cells for 2 days.
  d. Add 4 mL DMEM-media supplemented with 10% FCS + 1% Penicillin/Streptomycin.
  e. Add 750 μL feeder media 1 and 750 μL feeder media 2.
  f. Add 50 ng (final concentration 14.4 μM) murine recombinant macrophage colony-stimulating factor (M-CSF).
  g. Incubate the cells for 4 days.
  h. Replace the media by 10 mL DMEM media supplemented with 10% FCS + 1% Penicillin/Streptomycin.
  i. Incubate the cells for 1 day.
  j. Wash the plates one time with PBS.
  k. Detach the adherent macrophages by scraping with a cell scraper and use them for further experiments.

**CRITICAL:** It is important that the macrophages are in a good condition before starting the ADCP assay. Otherwise, their phagocytic activity will be impaired.

**CRITICAL:** After plating out the macrophages for further experiments, incubate them for 24 h for recovering before proceeding with the ADCP.

#### Preparation working with primary human macrophages

**Timing: at least 4 weeks**

4. Produce feeder media for later supply of differentiating monocytes.
  a. Plate out the murine fibroblast cell line L-929 cells on a 10 cm dish in non-supplemented RPMI 1640 media.
    ***Note:*** The conditioned media of L-929 cells contains growth factors, cytokines and others, which are particularly suitable for the differentiation of macrophages. Therefore, we recommend to use the conditioned media of this certain cell line.
  b. When the cells are dense, change the media to fresh non-supplemented RPMI 1640 media.
  c. Incubate the L-9292 cells.
  d. Collect the media of the L-292 cell dishes.
    i. After 1 week (further referred as “feeder media 1”).
    ii. After 3 weeks (further referred as “feeder media 2”).
      ***Note:*** Do not change the media until collecting day after performing step b.
  e. Centrifuge the media for 5 min at 300 *g* at 20°C to collect remaining cells in a pellet.
  f. Pipet the supernatant to a new tube to discharge the remaining cells.
  g. Store the feeder media at −20°C until further use.
5. Isolate peripheral blood mononuclear cells (PBMCs) from buffy coats.
  ***Note:*** Buffy coats are generated by a blood bank from a 500 ml blood donation. The plasma - and erythrocyte fraction is withdrawn from the donation bag after centrifugation for clinical use. The leftover middle fraction, representing the buffy coat, is provided to be used for research purposes. Here, normally 0.3 to 1∗10^12^ leukocytes can be isolated.
  a. Add 15 mL Lymphoprep to a 50 mL SepMate tube.
  b. Dilute the buffy coats 1:1 in sterile DPBS at 20°C.
  c. Layer 20 mL of the diluted buffy coat on the top of the SepMate tube.
    ***Note:*** Pipet the fluid to the walls of the tube.
  d. Centrifuge the tubes at 1200 *g* for 15 min at 20°C.
  e. Harvest the PBMCs by pouring the entire top layer in a new 50 mL falcon tube.
  f. Wash the cells three times with 50 mL DPBS with centrifugation at 300 *g* for 8 min at 4°C.
  g. Re-suspend the cells in 12 mL MACS buffer (4°C).
  h. Transfer the cells into a 15 mL tube.
  i. Centrifuge at 300 *g* for 8 min at 4°C and discard the supernatant.
  j. Add 200 μL CD14 anti-human magnetically labeled MicroBeads.
  k. Add 800 μL of MACS buffer (4°C).
  l. Perform magnetic separation on a flow cytometer (e.g., MacsQuant X flow cytometer with Miltenyi Biotec CD14 human microbead isolation protocol).
  m. Re-suspend CD14^+^ cells in non-supplemented RPMI 1640 media immediately.
6. Differentiate the monocytes into M2-like macrophages.
  a. Plate out 1 × 10^6^ CD14^+^ cells in 2 mL non-supplemented RPMI 1640 media per well on a 12 well plate.
  b. Add 10 ng/mL (final concentration 0.2 nM) M-CSF on day 1, day 3 and day 5 without changing the medium.
    ***Note:*** If you want to evaluate changes in polarization of the macrophages under treatment, add your reagent also on day 1, day 3 and day 5 to the macrophages.
  c. Scrape off the cells on day 7 for further use.
    **CRITICAL:** If you want to evaluate changes in phagocytosis due to changed polarization of the macrophages, you have to add your treatment while you are differentiating the monocytes into macrophages.
    **CRITICAL:** After plating out the macrophages for further experiments, incubate them for 24 h for recovering before proceeding with the ADCP.

#### Preparation working with cell lines

**Timing: at least 1 week**

7. Expand the macrophages.
  a. Thaw a frozen cell stock.
  b. Plate out the macrophages on a corning 10 cm dish in 10 mL media (media type see Table 1).

**Table 1:** Media for different cell types

Cell type	Media
J774A.1 cell line	DMEM media supplemented with 10% FCS and 1% penicillin-streptomycin
Primary human macrophages	RPMI 1640 media supplemented with 10% FCS
Primary murine macrophages	RPMI 1640 media supplemented with 10% FCS
hMB cells	DMEM media supplemented with 10% FCS and 1% penicillin-streptomycin
Primary CLL patient cells	RPMI 1640 media supplemented with 10% FCS
  c. Let them expand for at least 1 week to recover after thawing.
  d. If the plates get dense (cell confluence of more than 80%), split the cells but keep at least 1 × 10^4^ cells/mL.
  e. Check under the microscope if the macrophages are in a good condition.

**CRITICAL:** It is important that the macrophages are in a good condition before starting the ADCP assay. Otherwise, their phagocytic activity will be impaired. For a description and picture examples of macrophages in a good and a bad condition, see also “problem 1” in the section “troubleshooting”.

8. Expand the lymphoma cells.
  ***Note:*** In this approach the humanized-MYC/BCL2 (hMB) double-hit lymphoma cell line^9^ and primary CLL patient cells have been used. Several other cell lines or primary B cells might be used. However, an adaption of the measurement protocol might be needed. A clustering of the B cells has to be excluded for reliable flow cytometer measurement.
  a. Thaw a frozen cell stock.
  b. Plate out the lymphoma cells on a 10 cm dish in 10 mL media (media type see Table 1).
  c. Let them expand for at least 3 days to recover after thawing.

#### Tempering media for ADCP assays

**Timing: 1 h**

9. Warm up media to 37°C.

**CRITICAL:** Macrophages are sensitive to temperature changes, which could influence their phagocytic behavior. Therefore, longer times in cold areas or the addition of cold media must be avoided.

***Note:*** The media of lymphoma cells on day 2 of ADCP assay should be warmed up before use also.

## Key resources table

**Table:** 

REAGENT or RESOURCE	SOURCE	IDENTIFIER
**Antibodies**		

Alemtuzumab 10 mg/mL (anti-CD52 antibody)	MabCampath	NDC code 58468-0357-3
Daratumumab 120 mg/mL (anti-CD38 antibody)	Janssen-Cilag International N.V.	EMEA/H/C/004077
Obinutuzumab 25 mg/mL (anti-CD20 antibody)	Roche Registration Limited	EMEA/H/C/002799

**Chemicals, peptides, and recombinant proteins**		

Ammonium-chloride-potassium (ACK) lysis buffer	Thermo Fisher Scientific	Cat#A1049201
Gibco DMEM	Thermo Fisher Scientific	Cat#11574486
Fetal calf serum (FCS)	Thermo Fisher Scientific	Cat#15343681
Lymphoprep	STEMCELL Technologies	Cat#07801
Macrophage colony-stimulating factor (M-CSF), recombinant human	Thermo Fisher Scientific	Cat#PHC9501
M-CSF, recombinant mouse	Thermo Fisher Scientific	Cat#PMC2044
MACS separation buffer	Miltenyi Biotec	Cat#130-091-221
Microbeads CD14 human	Miltenyi Biotec	Cat#130-050-201; RRID:AB_2665482
Gibco penicillin-streptomycin (10,000 U/ml)	Thermo Fisher Scientific	Cat#11548876
Gibco RPMI 1640	Thermo Fisher Scientific	Cat#15364138
SepMate tube	Thermo Fisher Scientific	Cat#15654468

**Experimental models: Cell lines**		

Mouse: humanized-MYC/BCL2 double-hit lymphoma cell line (hMB), strain 102	N\A	Leskov et al. Rapid generation of human B-cell lymphomas via combined expression of Myc and Bcl2 and their use as a preclinical model for biological therapies. Oncogene 32, 1066–1072 (2013).^9^
Mouse: J774A.1 (macrophage cell line, origin: ascites of an adult, female mouse with reticulum cell sarcoma)	ATCC	Cat#TIB-67; RRID:CVCL_0358
Mouse: L-929 (fibroblast cell line)	DSMZ	Cat#ACC-2; RRID:CVCL_0462

**Experimental models: Organisms/strains**		

Mouse: wild-type C57BL/6J (male or female, age: 8–18 weeks)	Jackson Laboratory	Cat#000664; RRID:IMSR_JAX:000664
Mouse: wild-type NOD.Cg-Prkdc^scid^ Il2rg^tm1Wjl^/SzJ (NSG) (male or female, age: 8-18 weeks)	Jackson Laboratory	Cat#005557/NSG; RRID:IMSR_JAX:005557

**Software and algorithms**		

MACSQuantify	Miltenyi Biotec	https://www.miltenyibiotec.com

**Other**		

MACSQuant VYB flow cytometer	Miltenyi Biotec	Cat#130-096-116

## Step-by-step method details

### Culturing of macrophages for ADCP assay

**Timing: 25 h**

Macrophages are plated out in 96 well plates with 100 μL media/well. The number of macrophages needed is depending on macrophage type and written down in Table 2. Include at least five technical replicates per condition (= 10 wells/condition).

***Note:*** For ADCP assays with pretreatment of the macrophages or lymphoma cells, see step “pretreatment of macrophages” or “pretreatment of lymphoma cells”.

**Figure 1:**
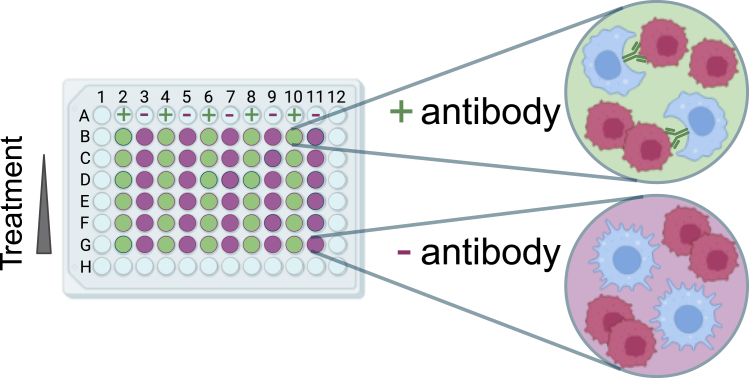
ADCP assay layout using a 96-well plate Blue wells (column 1 and 12 and row A and H): filled with sterile ware. Green wells (column 2, 4, 6, 8, 10): antibody-treated co-culture of macrophages and lymphoma cells. Purple wells (column 3, 5, 7, 9, 11): co-culture of macrophages and lymphoma cells without antibody treatment. Row B: control row without addition of the treatment of interest. Row C-G: Wells treated with the compound of interest with increasing concentration per row.

1. Ensure the macrophages are in a good condition.
  a. Regard the macrophages on cell culture dishes under the microscope.
2. Plate out the macrophages.
  a. Scrape off the macrophages with a cell scraper.
  b. Count the cells and define the viability of the macrophages.
  c. Centrifuge the needed number of viable macrophages (see Table 2) for 5 min at 300 *g* at 20°C.
  d. Discard the supernatant.
  e. Re-suspend the cells in fresh pre-warmed macrophage media (see Table 1) (needed volume: 100 μL/well).
  f. Plate out 100 μl/well cell suspension on a 96 well plate (see also Figure 1).
    i. Do not use the outer wells of the cell plate.
    ii. Fill up the outer wells with 250 μL liquid (e.g., water).

**CRITICAL:** The final measurement is performed with total cell count /μL. Therefore, an evaporation of media has to be prohibited. To ensure avoidance of evaporation the outer wells of the plates are filled with fluid.

3. Incubate the plates for 24 h to let the macrophages adhere.

**Table 2:** Cell seeding number for various sources of macrophages

Macrophage cell type	Macrophage number/well	Lymphoma cell number /well
Murine macrophage cell line J774A.1	1 × 10^4^	1.5 × 10^5^
Primary human macrophages	2 × 10^4^	3 × 10^5^
Primary murine macrophages	5 × 10^4^	1.5 × 10^5^

### Preparation of co-culture treatment

**Timing: 30 min**

In this section dilution series of treatment compound and antibody dilution are prepared.

***Note:*** The treatment of the co-culture should be performed as quick as possible after addition of lymphoma cells to the macrophages as phagocytosis reaction will begin already after the addition. Therefore, all reagents should be prepared before adding the lymphoma cells to the co-culture.

4. Prepare a dilution series of your treatment compound.
  ***Note:*** The final volume needed is 25 μL/well (needed: at least five replicates = 10 wells /condition).
  a. Dilute your reagent in macrophage media (see Table 1).
  b. Produce two further dilution steps of your reagent (e.g., 1/2 and 1/10).
5. Prepare antibody solution.
  a. Dilute your antibody (see Table 3) in macrophage media (see Table 1).

***Note:*** needed amount: 25 μL/well for half of the co-culture wells

**Table 3:** Antibody type and concentration for different macrophage types

Macrophage type	Antibody	Final concentration
J774A.1 cell line	Alemtuzumab	10 μg/mL
Primary human macrophages	Daratumumab	10 μg/mL
Primary murine macrophages	Daratumumab	10 μg/mL

### Co-culturing of lymphoma cells with macrophages

**Timing: 1 h**

In this section lymphoma cells are added to the macrophages on the 96 well plates.

6. Plate out the lymphoma cells.
  a. Count and define the viability of the lymphoma cells.
  b. Centrifuge the needed number of viable lymphoma cells (see Table 2) for 5 min at 300 *g* at 20°C.
  c. Discard the supernatant.
  d. Re-suspend the cells in fresh pre-warmed media for lymphoma cells (see Table 1) (needed volume: 100 μL/well).
  e. Add 100 μl/well lymphoma cell suspension to every well containing macrophages (see also Figure 1).
    i. Do not use the outer wells of the cell plate.

### Treatment of the co-culture

**Timing: 19 h**

In this section treatment of the co-culture and incubation during antibody-dependent cellular phagocytosis is performed.

7. Add 25 μL/well macrophage media (see Table 1) to at least five replicates (= 10 wells) = control replicates (see also Figure 1).
8. Add 25 μL/well treatment solution to at least five replicates (= 10 wells) (see also Figure 1).
  a. Do so for every treatment concentration you have prepared.

**CRITICAL:** in case of pretreatment of macrophages or lymphoma cells, do not add treatment solution to the co-culture. Instead, add 25 μL/well macrophage media (see Table 1) to the treatment wells.

9. Add 25 μL/well macrophage media (see Table 1) to every second well (see also Figure 1).
10. Add 25 μL/well antibody solution to every other second well (see also Figure 1).
11. Incubate the plates for 18 h.

**CRITICAL:** the final volume of each well has to be 250 μL.

### FACS measurement of non-phagocytosed lymphoma cells

**Timing: 1 h**

In this section the number of non-phagocytosed lymphoma cells is determined by FACS measurement of the co-cultures.

12. Cool down the cell plates for 10 min at 4°C.

**CRITICAL:** During this step, remaining antibody-labeled lymphoma cells will be detached from the cell surface of macrophages and lymphoma cell aggregates will be dissolved. Without cooling down the cells before performing FACS measurement, the results will be instable as the plate cools down over time during measurement and the attached and aggregated lymphoma cells will be solved causing an increasing absolute lymphoma cell count over time.

13. Prepare the flow cytometer for the measurement.
  ***Note:*** The flow cytometer measurement by which this protocol was implemented was performed with MACSQuant VYB (Miltenyi, Germany). Other flow cytometer may lead to less stable results due to different techniques of mixing or measurement (see also troubleshooting).
  ***Note:*** You need a flow cytometer with the possibility to measure absolute cell counts. If an absolute counting mode is not applicable, you may use counting beads (e.g., CountBright Counting Beads from Thermo Fisher Scientific) for quantification. However, an adaption of the measurement protocol might be needed.
  **CRITICAL:** Your flow cytometer must have a maximal fault tolerance of 5% for absolute cell count. Otherwise, the measurement is not reliable.
  a. Use a probe of pure lymphoma cells to prepare the gating.
    i. Gate 1: forward light scatter/sideward light scatter (FSC/SSC) of the lymphoma cells.
    ii. Optional (if your lymphoma cells are fluorescent): Gate 2: Fluorophore-positive cells of Gate 1.
  b. If your flow cytometer is able to mix cells, adjust the mixing mode to single well mixing before each measurement.
    i. If your flow cytometer is not able to mix your probes, pipet up and down each well several times before measurement.
      ***Note:*** If your assay includes more than 30 wells, this step should be repeated with the remaining wells after measurement of maximal 30 wells.
      **CRITICAL:** Do not use a whole plate shaking mode before each measurement on your flow cytometer. This will lead to displace of the lymphoma cells to the borders of the wells and will confound the measurement of the absolute cell count /μL (see also troubleshooting).
  c. Adjust measured volume to 25 μL.
  d. Adjust readout to absolute cell count /μL of Gate 1 (Gate 2 in case of using fluorophore-positive cells).
  e. Adjust the measurement alignment in the direction, that not one condition is measured in total but one well of each condition after another.
14. Measure your 96 well plate with the above-mentioned parameters.

### Calculate antibody-dependent cellular phagocytosis rate

**Timing: 15 min**

**Figure 2:**
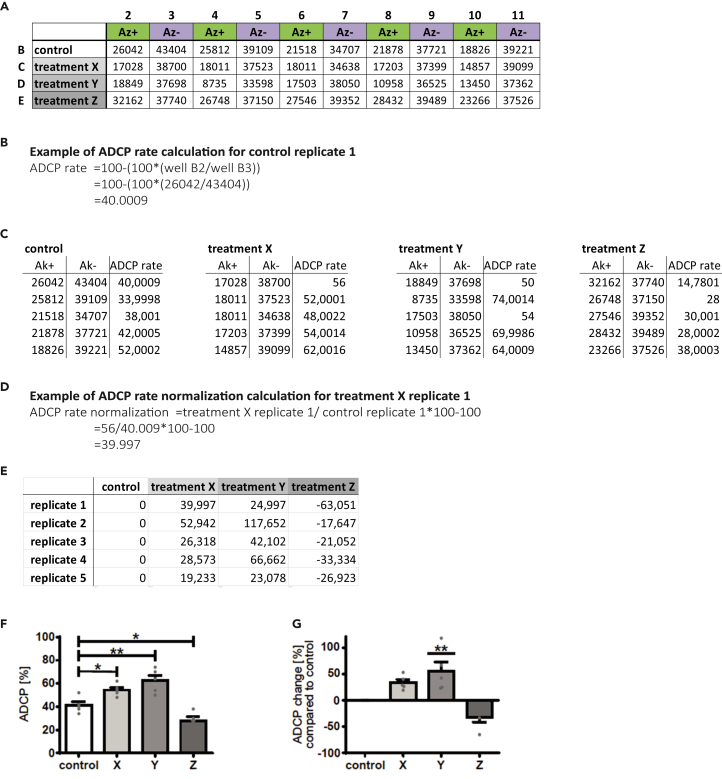
Fictive readout, calculation, and bar plot presentation of an ADCP assay (A) Measured absolute lymphoma cell count presented in a 96 well layout. Treatment X-Z representing different treatment conditions. (B) Example of ADCP rate calculation based on values of well B2 and B3 of (A). (C) Calculated ADCP rates based on values of (A). (D) Example of ADCP rate normalization of replicate 1 of treatment X. (E) Calculated normalized ADCP rates based on values of A). (F) Bar plot presentation of ADCP rates with performed One-way ANOVA for significance testing. ∗*p* < 0.05; ∗∗*p* < 0.01. (G) Bar plot presentation of ADCP change with performed One-way ANOVA for significance testing. ∗∗*p* < 0.01.

In this section the ADCP rate is calculated out of the measured non-phagocytosed lymphoma cells in antibody treated wells in comparison to wells with our antibody treatment. Figure 2 visualizes the calculation steps and following possible graphical presentation.

15. Calculate the ADCP rate with the following formula: 100-(100∗(total cell count antibody-treated well/total cell count antibody-untreated well)).
  a. Use the total amount of fluorophore-positive cells /μL if applicable.
16. If wanted, calculate the ADCP change between the treated and the control cells with the following formula: Treatment well X_[1-n]_/ control well X_[1-n]_∗100-100.
  a. X = replicate; X_1_ = replicate 1, X_2_ = replicate 2, X_n_ = replicate n.
  b. Repeat the calculation for every replicate.

***Note:*** Macrophages are very sensitive to minimal changes in their environment. Therefore, their basal phagocytosis rate often differs between repetitive experiments and can obscure effects of the treatment. An adjustment to the basal phagocytosis rate by calculating the ADCP change can be helpful for deception avoidance.

### Pretreatment of macrophages

**Timing: 31 h**

To evaluate the isolated effect of treatment on the phagocytic activity of macrophages, this section elucidates the pre-treatment of macrophages before adding them to the co-culture.

17. Ensure the macrophages are in a good condition by checking the morphology of the cells using a light microscope.
18. Plate out the macrophages.
  a. Scrape off the macrophages with a cell scraper.
  b. Count the cells and define the viability of the macrophages.
  c. Centrifuge the needed number of macrophages (see Table 2) for 5 min at 300 *g* at 20°C.
  d. Discard the supernatant.
  e. Re-suspend the cells in fresh pre-warmed macrophage media (see Table 1) (100 μL/well).
  f. Plate out 100 μL/well cell suspension on a 96 well plate.
    i. Do not use the outer wells of the cell plate.
    ii. Fill up the outer wells with 250 μL fluid.

**CRITICAL:** The final measurement is performed with total cell count /μL. Therefore, an evaporation of media has to be prohibited. To ensure avoidance of evaporation the outer wells of the plates are filled with fluid.

19. Let the cells adhere for at least 5 h.
20. Add 25 μL/well macrophage media (see Table 1) as a control to at least five replicates (n = 10 wells) = control replicates.
21. Add 25 μL/well treatment solution to at least five replicates (n = 10 wells).
  a. Do so for every treatment concentration you have prepared.
22. Incubate the plates for 24 h.
23. Wash the macrophages three times.
  **CRITICAL:** Do not touch the bottom of the wells as this would detach the macrophages and have great influence on the following steps.
  a. Discard the media by pipetting without touching the well bottom.
  b. Add 100 μL/well macrophage media (see Table 1).
  c. Repeat these steps two more times.
24. Continue with step 6.

### Pretreatment of lymphoma cells

**Timing: 26 h**

To evaluate the isolated effect of your treatment on the phagocytic activity of macrophages due to changes in the lymphoma cells with possible changed susceptibility toward phagocytosis, this section elucidates the pre-treatment of lymphoma cells before adding them to the co-culture.

25. Plate out 2.4 × 10^6^ lymphoma cells in 1.5 mL/well on a 6-well plate on the same day you are plating out the macrophages for the ADCP-assay (media see Table 1).
26. Add 250 μL lymphoma media (see Table 1) to one well as a control experiment.
27. Add 250 μL treatment solution to one well.
  a. Do so for every treatment concentration you have prepared.
28. Incubate the plates for 24 h.
29. Wash the lymphoma cells.
  a. Transfer the cells into tubes.
  b. Centrifuge the cells for 5 min at 300 *g* at 20°C.
  c. Discard the supernatant.
  d. Re-suspend the cells in 1 mL media (see Table 1).
  e. Repeat steps b–d two more times.
30. Continue with step 6.

## Expected outcomes

This protocol can be used to identify the phagocytic activity of different macrophage cell types as well as the amount of phagocytosis of different lymphoma cells. The influence of agents on the phagocytic activity of macrophages and on the susceptibility of lymphoma cells toward macrophage phagocytosis can be determined. The use of macrophages and lymphoma cell from cell line, murine, and human origin is depicted.^1^^,^^3^^,^^7^^,^^8^

Furthermore, the determination of the leading cell type causing the changes in observed phagocytic activity is feasible.

## Limitations

To produce robust results performing ADCP assays, a precise way of pipetting and working under equal conditions has to be performed.

Macrophages are sensitive to any environmental changes. Changes in their cultivation or the temperature of their media can already have an influence on their phagocytic activity. Therefore, strict equal conditions have to be met. Different kind of surfaces and plastic can influence their behavior and phagocytic activity also. Therefore, the used cell culture materials should be tested for every macrophage type used before performing the assays.

The lymphoma cells have to be usable for flow cytometer analysis performing antibody treatment. Several lymphoma cells lines, e.g., OSU, are building broad clusters under antibody treatment possibly causing flow cytometer plugging and leading to unreliable results. Therefore, every lymphoma cell line should be tested toward clustering before performing the assays.

Not every lymphoma cell is carrying epitopes for human clinical antibodies. If so, they are not applicable for the here mentioned antibodies. It has to be evaluated, if there are alternative antibodies available for this purpose.

Using primary cells, a particular attention has to be drawn to their viability and behavior. Primary murine macrophages need at least 24 h to recover after any interventions. Therefore, the cells should rest after differentiation for one day before starting the ADCP assay. Primary human macrophages should also recover for 24 h. Primary lymphoma cells often depend on surrounding immune cells and might therefore have a quick decrease in viability cultivating them in mono-culture or in co-culture only with macrophages. Therefore, primary lymphoma cells should be used as quick as possible after receipt.

Attention should be drawn to the different toxicity of reagents toward different macrophage or lymphoma cell types. Therefore, a new toxicity evaluation should be performed for every new cell type used. There should be reached a viability of at least of 95% of both cell types used to exclude effects on phagocytosis driven by cell damage / cell death signaling.

To measure the absolute cell count in a determined volume, a flow cytometer with the ability to measure absolute cell count with a maximal fault tolerance of 5% has to be used. If an absolute counting mode is not applicable, one may use counting beads (e.g., CountBright Counting Beads from ThermoFisher) for quantification. However, this has not been tested in the offered protocol and an adaption of the measurement protocol might be needed.

The flow cytometer has to be able to mix the wells individually by pipetting and not by an orbital shaking mode of the whole plate as this is leading to unstable results.

Under incubation a clustering of especially antibody-labeled cells can appear. This clustering is decreasing under temperature decrease during measurement possibly leading to unstable results. Therefore, cell plates should be cooled down for at least 10 min (equal time period for each measurement) before starting the measurement.

The co-culture used for ADCP assays is a simplified model of phagocytosis in lymphoma. The lymphoma microenvironment consists of a broader range of immune and bystanding cells, which may have an influence on macrophage function and lymphoma cells, whereby the phagocytic activity of macrophages and the phagocytosis of lymphoma cells might be changed. Therefore, the results of the *in vitro* assays are not transferrable without *in vivo* validation.

## Troubleshooting

### Problem 1

Macrophages are not growing well or are in a bad condition (related to Preparation 3.k, 7.e and step-by-step method details 1.a, 17).

**Figure 3:**
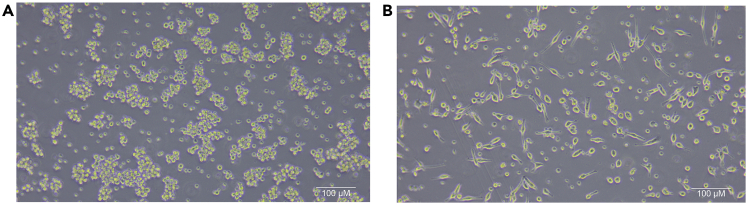
Possible appearances of macrophages (A) Macrophages in a bad condition. The cells cluster and appear as round small cells. (B) Macrophages in a good condition. The macrophages appear spread and with extensions.

If the macrophages are in a bad condition, they appear round and small under the microscope. If they are in a good condition, they appear spread and with extensions (Figure 3).

### Potential solution

• Macrophages need a certain rough underground to behave well and proliferate. We have observed, that all types of used macrophages in this manuscript behave the best on Corning plates. Therefore, just these plates (e.g., Falcon 100 mm TC-treated cell culture dish, Corning) should be used.
• Macrophages need cell-cell contacts and therefore a certain density for behaving well and expanding. Plate out the cells in a density of at least 1 × 10^4^ cells/mL.

### Problem 2

High variability of technical replicates (related to step-by-step method details 14).

### Potential solution

• Take strict control of equal conditions especially for the macrophage culture. Macrophages react very sensitive to even small changes in their environment.
• Perform very precise pipetting. For the ADCP assay, total cell amounts are measured. Changes in the total volume of your sample or the cell amount /well of macrophages or lymphoma cells have a great influence on the final measurement and the phagocytosis reaction. To optimize the pipetting, multi-channel pipets can be used.
• Cool down your 96 well plate for at least 10 min before the measurement. Remaining antibodies or lymphoma-macrophage binding can lead to lymphoma cell clustering. By concomitant cooling of the plate during measurement, this clustering is decreased and can lead to differing absolute cell count over the proceeding measurement.

### Problem 3

The results in the outer wells of the 96 well plate differ from the rest of the wells (related to step-by-step method details 14).

As this assay is measuring the absolute cell count in a determined volume, an evaporation of media is changing the result. Without a rescue against evaporation in the outer wells, this is taking place.

### Potential solution

• Do not use the outer wells of a 96 well plate for your assay.
• Fill the outer wells instead with a fluid to build a rescue line against evaporation.

### Problem 4

**Figure 4:**
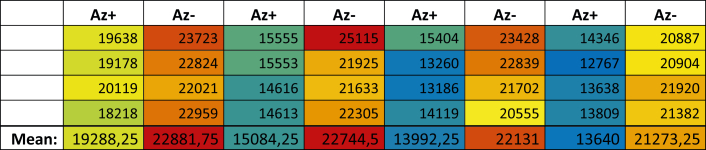
Example of decreasing cell count during measurement by using orbital shaking mode Az- = wells without antibody treatment, Az+ = wells with antibody treatment. The total cell amount is displayed. The color is representing the cell count (red: highest cell count, blue: lowest cell count). Note: wells with antibody treatment have a lower cell amount in total due to increased phagocytosis of antibody-labeled cells by macrophages.

The measured cell count is constantly decreasing during measurement (related to step-by-step method details 14). Example shown in Figure 4.

### Potential solution

• Do not use a mixing mode of flow cytometer, which is performing an orbital shaking of the whole plate before each measurement. The fluid cells are pushed toward the borders of the wells and are not reached by the needle for measurement any more.
• Use a mixing mode, which is mixing up each well directly before measurement by pipetting up and down the fluid.

## Resource availability

### Lead contact

Further information and requests for resources and reagents should be directed to and will be fulfilled by the lead contact, Anna Beielstein (anna.beielstein@uk-koeln.de).

### Technical contact

Technical questions on executing this protocol should be directed to and will be answered by the technical contact, Anna Beielstein (anna.beielstein@uk-koeln.de) and Christian Pallasch (christian.pallasch@uk-koeln.de).

### Materials availability

This study did not generate new unique reagents.

### Data and code availability

This study did not generate or analyze datasets or code.

## Acknowledgments

We are indebted to our patients who contributed blood samples to this study. This work was funded by the 10.13039/501100001659Deutsche Forschungsgemeinschaft (DFG, German Research Foundation) KFO28 and SFB1530 (SFB-Geschaeftszeichen – 455784452, project B02). C.P.P. was supported by the “Foerderprogramm Nachwuchsforschungsgruppen NRW 2015–2021,” CAP Program of the Centre for Molecular Medicine Cologne, the “Deutsche Jose-Carreras Leukaemiestiftung e.V.” (DJCLS 07R/2021), and a research grant by 10.13039/100005564Gilead Sciences. A.C.B. was supported by Studentische Forschungsfoerderung/Begabtenfoerderung” of Koeln Fortune program of the medical faculty of University of Cologne. The graphical abstract and Figure 1 have been created in BioRender (Beielstein, A. [2025]; https://BioRender.com/kbj5epv, https://BioRender.com/zab6npa).

## Author contributions

Study design, A.C.B. and C.P.P.; data analysis and acquisition, A.C.B. and S.B.; samples and annotation, C.P.P.; study supervision and funding, C.P.P.; manuscript preparation, A.C.B. and C.P.P.

## Declaration of interests

The authors declare no competing interests.

## References

[1] Beielstein A.C., Izquierdo E., Blakemore S., Nickel N., Michalik M., Chawan S., Brinker R., Bartel H.-H., Vorholt D., Albert L. (2024). Macrophages are activated toward phagocytic lymphoma cell clearance by pentose phosphate pathway inhibition. Cell Rep. Med..

[2] Coiffier B., Thieblemont C., Van Den Neste E., Lepeu G., Plantier I., Castaigne S., Lefort S., Marit G., Macro M., Sebban C. (2010). Long-term outcome of patients in the LNH-98.5 trial, the first randomized study comparing rituximab-CHOP to standard CHOP chemotherapy in DLBCL patients: a study by the Groupe d’Etudes des Lymphomes de l’Adulte. Blood.

[3] Pallasch C.P., Leskov I., Braun C.J., Vorholt D., Drake A., Soto-Feliciano Y.M., Bent E.H., Schwamb J., Iliopoulou B., Kutsch N. (2014). Sensitizing protective tumor microenvironments to antibody-mediated therapy. Cell.

[4] Beielstein A.C., Pallasch C.P. (2019). Tumor Metabolism as a Regulator of Tumor-Host Interactions in the B-Cell Lymphoma Microenvironment-Fueling Progression and Novel Brakes for Therapy. Int. J. Mol. Sci..

[5] Colegio O.R., Chu N.-Q., Szabo A.L., Chu T., Rhebergen A.M., Jairam V., Cyrus N., Brokowski C.E., Eisenbarth S.C., Phillips G.M. (2014). Functional polarization of tumour-associated macrophages by tumour-derived lactic acid. Nature.

[6] Liu D., Chang C., Lu N., Wang X., Lu Q., Ren X., Ren P., Zhao D., Wang L., Zhu Y. (2017). Comprehensive Proteomics Analysis Reveals Metabolic Reprogramming of Tumor-Associated Macrophages Stimulated by the Tumor Microenvironment. J. Proteome Res..

[7] Izquierdo E., Vorholt D., Blakemore S., Sackey B., Nolte J.L., Barbarino V., Schmitz J., Nickel N., Bachurski D., Lobastova L. (2022). Extracellular vesicles and PD-L1 suppress macrophages, inducing therapy resistance in TP53-deficient B-cell malignancies. Blood.

[8] Barbarino V., Henschke S., Blakemore S.J., Izquierdo E., Michalik M., Nickel N., Möllenkotte I., Vorholt D., Müller L., Brinker R. (2020). Macrophage-Mediated Antibody Dependent Effector Function in Aggressive B-Cell Lymphoma Treatment is Enhanced by Ibrutinib via Inhibition of JAK2. Cancers (Basel).

[9] Leskov I., Pallasch C.P., Drake A., Iliopoulou B.P., Souza A., Shen C.-H., Schweighofer C.D., Abruzzo L., Frenzel L.P., Wendtner C.M. (2013). Rapid generation of human B-cell lymphomas via combined expression of Myc and Bcl2 and their use as a preclinical model for biological therapies. Oncogene.

